# Supplementary material for: Steep-slope vertical-transport transistors built from sub-5 nm Thin van der Waals heterostructures
Source: Nat Commun. 2024 Feb 7;15:1138. doi: 10.1038/s41467-024-45482-x (PMC10850082; doi:10.1038/s41467-024-45482-x)
Supplement: Supplementary file 1 — Supplementary Information [file 41467_2024_45482_MOESM1_ESM.pdf]

Supplementary Information for

Steep-Slope Vertical-Transport Transistors Built from sub-5 nm Thin van der Waals Heterostructures

Qiyu Yang[1], Zheng-Dong Luo[1,2,*], Huali Duan[3], Xuetao Gan[4,*], Dawei Zhang[5,6], Yuewen Li[2], Dongxin Tan[1], Jan Seidel[5,6], Wenchao Chen[3], Yan Liu[1,2*], Yue Hao[1] and Genquan Han[1,2]

[1]State Key Discipline Laboratory of Wide Band Gap Semiconductor Technology, School of Microelectronics, Xidian University, Xi’an 710071, China

[2]Hangzhou Institute of Technology, Xidian University, Hangzhou 311200, China

[3]ZJU-UIUC Institute, International Campus, Zhejiang University, Haining 314400, China

[4]Key Laboratory of Light Field Manipulation and Information Acquisition, Ministry of Industry and Information Technology, and Shaanxi Key Laboratory of Optical Information Technology, School of Physical Science and Technology, Northwestern Polytechnical University, Xi’an 710129, China

[5]School of Materials Science and Engineering, UNSW Sydney, Sydney NSW 2052, Australia

[6]ARC Centre of Excellence in Future Low-Energy Electronics Technologies (FLEET), UNSW Sydney, Sydney NSW 2052, Australia

*Email: zhdluo@xidian.edu.cn; xuetaogan@nwpu.edu.cn; xdliuyan@xidian.edu.cn

Contents

Supplementary Note 1: Fabrication process for the TS-VTFETs 3
Supplementary Note 2: Thickness characterization of the TS-VTFET 4
Supplementary Note 3: Band profile analysis of the VTFETs 5
Supplementary Note 4: Temperature dependent output characteristics of the VTFETs 8
Supplementary Note 5: Transfer characteristics of different fabricated transistors 9
Supplementary Note 6: Discussion on the contribution of the lateral conducting channel to the overall device transport properties 10
Supplementary Note 7: Discussion of the device behaviour of a VTFET with the gate electrode confined within the junction area 13
Supplementary Note 8: Microscopic structure of the Ag/TaO_x/TaO_y/TaO_x/Ag TS cell 15
Supplementary Note 9: Electrical properties of the TS cell 16
Supplementary Note 10: The electrode configuration effect on electrical properties of the TS cell 17
Supplementary Note 11: Topography of the fabricated Al_2O_3/HfO_2 substrate 18
Supplementary Note 12: Analysis of the electrical behaviours of the TS-VTFET during the current switching process 19
Supplementary Note 13: Voltage cycling endurance of the TS-VTFETs 22
Supplementary Note 14: Comparison of key figure-of merits of TS-VTFET with previous VTFETs 23
Supplementary Note 15: Comparison of key figure-of merits of TS-VTFET with previous reported emerging steep-slope transistors 24

Supplementary Note 1: Fabrication process for the TS-VTFETs

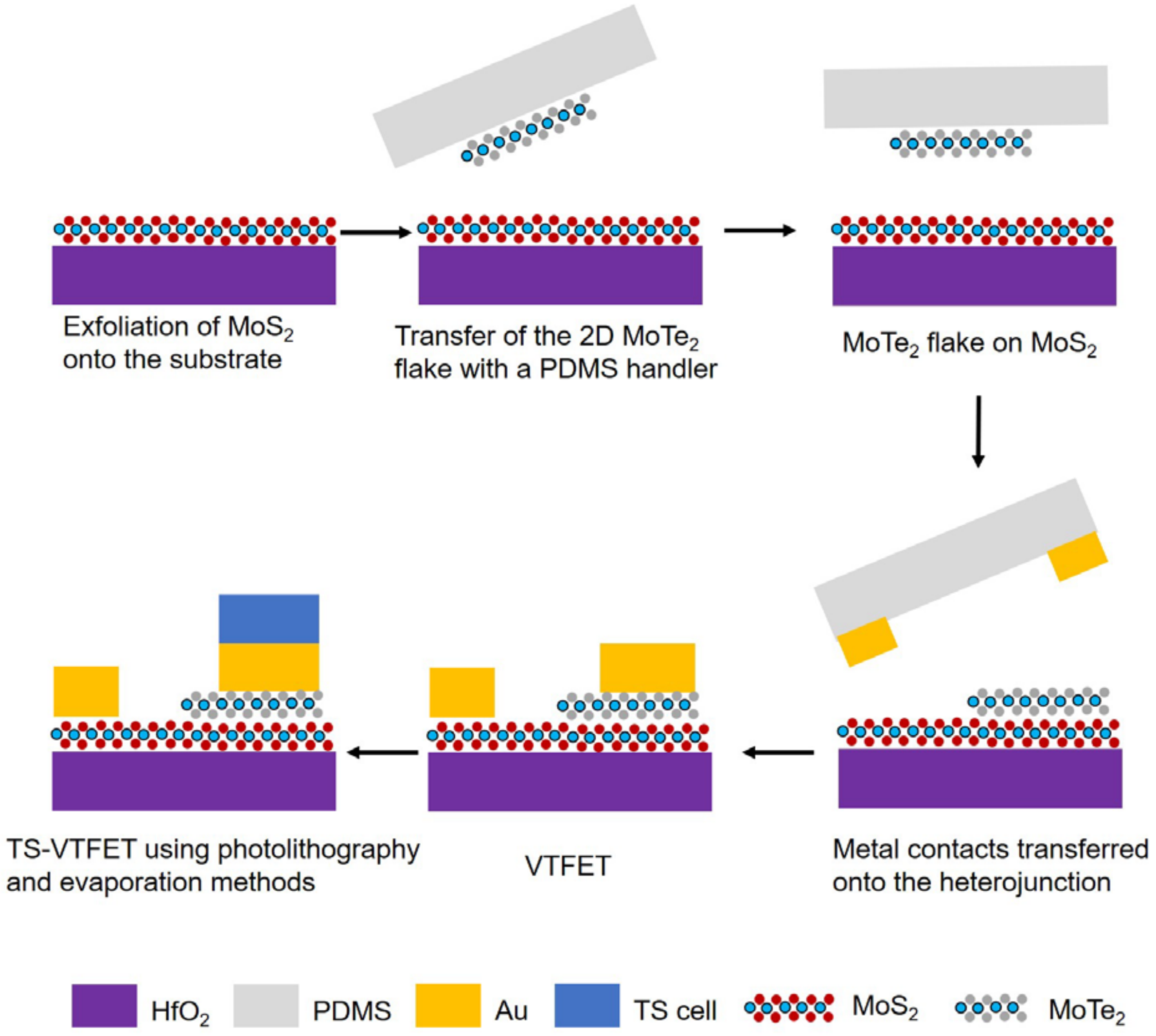

Supplementary Figure 1 | The fabrication flow of the TS-VTFET. The MoS_2 and $MoTe_2$ flakes of interest were transferred via PDMS support onto the substrate. To fabricate the source and drain contact, Au electrodes of photolithography pre-defined shape were transferred using PDMS onto the 2D flakes. The as-fabricated VTFETs were annealed at 300 °C in vacuum for 2 h. Next, a standard photolithography process was conducted to open the window for the TS cell on the drain terminal of the VTFETs. Finally, magnetron sputtering of alternating TaO_x/TaO_y/TaO_x and Ag layers was finally carried out to form the TS cell on the selected area.

Supplementary Note 2: Thickness characterization of the TS-VTFET

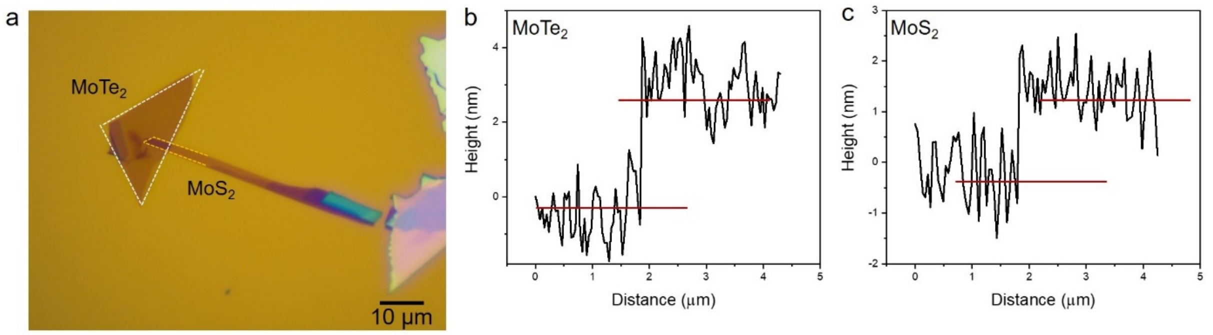

Supplementary Figure 2 | Optical image (a) and thickness measurement of the $MoS_2/MoTe_2$ heterostructure using AFM. The height for MoS_2 and $MoTe_2$ is ~3.2 nm (4 layers) and ~1.7 nm (2 layers), respectively. Electrical data of the TS-VTFET in the main text were taken from the presented device.

Supplementary Note 3: Band profile analysis of the VTFETs

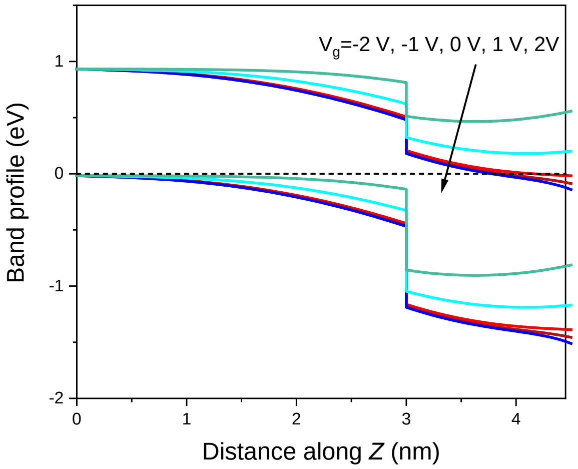

Supplementary Figure 3 | Band profiles of the VTFET under different gate biases (V_g = -2 V, -1 V, 0 V, 1 V, 2 V) at V_d = 0 V, where the dashed line represents the Fermi energy level.

To elucidate the operation mechanism of the proposed VTFET, the band profiles are calculated by using the finite difference (FD) method to solve the 2-D Poisson's equation. The geometric and material parameters including thickness T, bandgap E_g, electron affinity χ, relative permittivity ε_r, and electron/hole effective mass $m^*_{e(h)}$ of the corresponding layer are listed in Table 1[1-5]. The doping concentrations in p-type $MoTe_2$ and n-type MoS_2 are set as N_A = 2.0×10^{12} /cm^2 and N_D = 2.0×10^{12} /cm^2, which are estimated from the experimental results by $N_{A(D)} = \frac{1}{q}\frac{C_{HfO_2}C_{Al_2O_3}}{C_{HfO_2}+C_{Al_2O_3}} / (V_{th} - V_g)$ /[6].

Supplementary Table 1 | Geometric and material parameters of the VTFET.

Symbol	Quantity	$MoTe_2$	MoS_2	Al_2O_3	HfO_2
T	thickness	3 nm	1.5 nm	2.5 nm	12 nm
E_g	bandgap	0.94 eV [1]	1.37 eV [1]	8.8 eV [2]	5.7 eV [2]
χ	electron affinity	4.0 eV [3]	4.3 eV [4]	1.45 eV [2]	2.65 [2]
ε_r	relative permittivity	7 [5]	7 [5]	11 [2]	20 [2]
m_e^*	electron effective mass	$0.57m_0$ [4]	$0.49m_0$ [4]	/	/
m_h^*	hole effective mass	$0.70m_0$ [4]	$0.58m_0$ [4]	/	/

The Poisson's equation is given as follows,

$$-\nabla \cdot (\varepsilon_r \nabla \mathrm{V}) = (\mathrm{p} - \mathrm{n} - N_A + N_D)q/\varepsilon_0 \quad (1)$$

where V is the electric potential, ε_0 and ε_r are the vacuum permittivity and the relative permittivity of corresponding materials, $N_{D(A)}$ is donor(acceptor) density, and $n(p)$ is the electric potential related electron(hole) density. The electron density which can be calculated by the Fermi-Dirac integral is given below as an example, and the hole density can be obtained similarly.

$$n = N_{3D}\frac{2}{\sqrt{\pi}}F_{\frac{1}{2}}(\eta_F) \quad (2)$$

where $N_{3D} = 2(\frac{2\pi m_e^* k_B T}{h^2})^{3/2}$, $F_{\frac{1}{2}}(\eta_F) = \int_0^\infty \frac{\varepsilon^{1/2}d\varepsilon}{1+e^{(\varepsilon-\eta_F)}}$, $\eta_F = (E_{Fn} - E_C)/k_B T$, m_e^* is the electron effective mass, k_B is the Boltzmann constant, h is the Planck constant, and E_{Fn} denotes the electron quasi-Fermi level. The Schottky contact boundary condition is set at the bottom gate electrode, and ohmic boundary conditions are set at the source and drain electrodes.

The simulated band profiles along the vertical direction of the VTFET under different gate bias at V_d = 0 V is shown in Supplementary Figure 3. The results indicate that the bottom gate can modulate the band profile of MoS_2 and then further control the band alignment of the $MoTe_2/MoS_2$ VTFET. The band profiles under different V_g and V_d are shown in the main text Figure 2g and Supplementary Figure 4. The VTFET behaves like a gate-controlled *p-n* diode and tunnel diode as $V_d > 0$ V and $V_d < 0$ V, respectively. As $V_d > 0$, the VTFET works as a

conventional forward-biased *p*-*n* junction. As V_g = 1 V, there is a significant current flow in the device due to the diffusion of majority carriers from MoS_2 to $MoTe_2$ as in Supplementary Figure 4a. As V_g decreases, the current significantly decreases due to the exponential decrease of the majority carriers in MoS_2 as shown in Supplementary Figure 4b, 4c. As V_d < 0, the VTFET operates as a gate-controlled reverse-biased tunnel diode. As V_g = 1V, the electrons tunnel from the valence band of $MoTe_2$ to the conduction band of MoS_2 as shown in Supplementary Figure 4d, where the barrier width for the tunnelling of electrons is denoted by x_B. As V_g decreases while V_d remains unchanged, the overlap between the conduction band of MoS_2 and the valence band of $MoTe_2$ is reduced as in Supplementary Figure 4e. Within the energy window where the electrons can tunnel into MoS_2, the barrier width x_B increases since the band tilt in the diode is weakened as V_g decreases. As a result, the tunnelling current decreases. As V_g further decreases to the extent that there are no available states in MoS_2 to accommodate the electrons tunnelling from $MoTe_2$ as shown in Supplementary Figure 4f, the current will be impeded. Under this condition, a large onset tunnelling voltage of V_d (more negative) is required to present a high current level as shown in the main text Figure 2f of the manuscript.

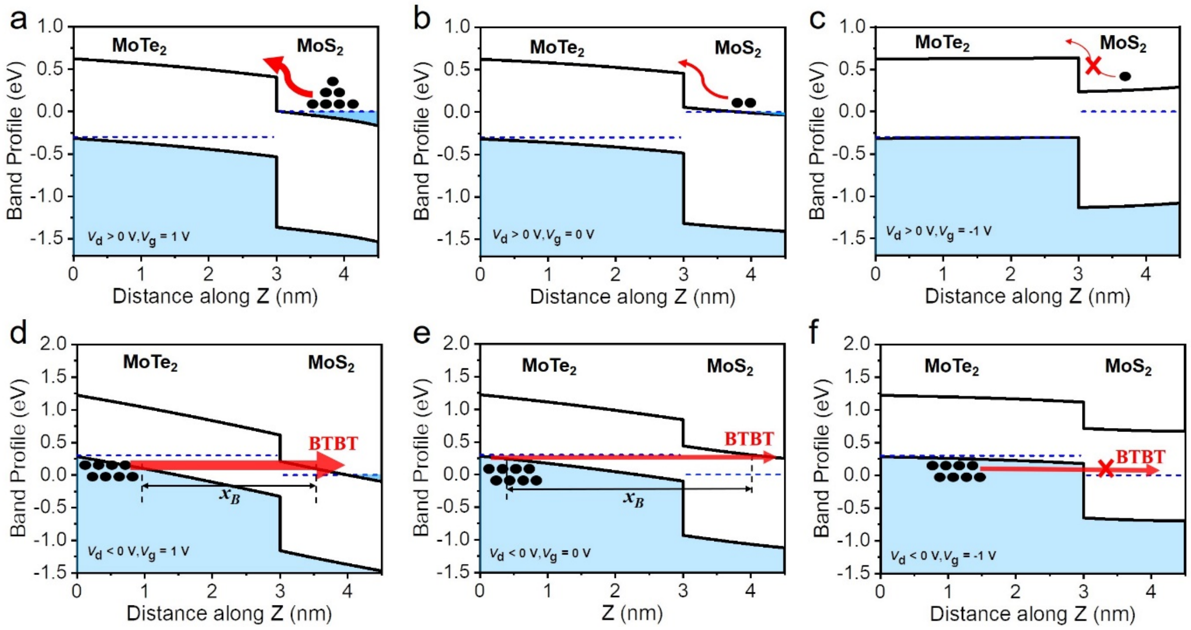

Supplementary Figure 4 | Calculated band profiles under different V_d and V_g of the $MoS_2/MoTe_2$ vdW heterostructure-based VTFET. As V_d > 0, the VTFET works as a conventional forward biased p-n diode: **a** V_g = 1V, **b** V_g = 0V, and **c** V_g = -1V. As V_d < 0, the VTFET operates as a gate-controlled reverse biased tunnel diode: **d** V_g = 1V, **e** V_g = 0V, and **f** V_g = -1V, where x_B represents the barrier width of electron tunnelling.

Supplementary Note 4: Temperature dependent output characteristics of the VTFETs

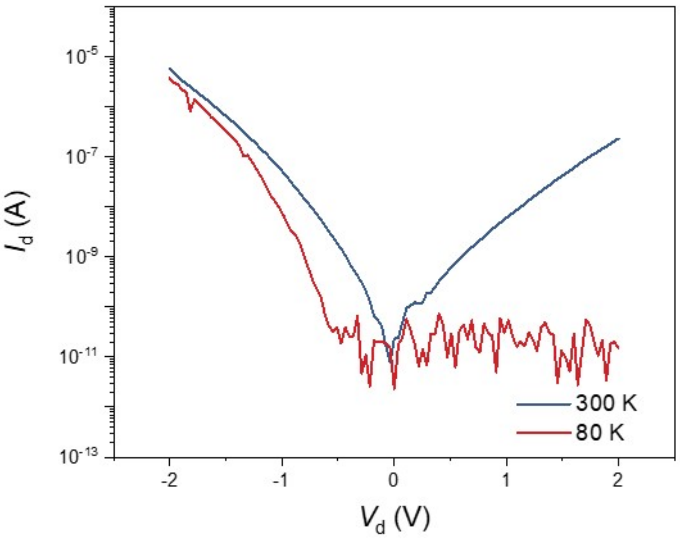

Supplementary Figure 5 | Output curves of the $MoS_2/MoTe_2$ heterojunction-based VTFETs under zero gate voltage at 300 K and 80 K, respectively. At the positive V_d region, the $MoS_2/MoTe_2$ heterojunction works as a conventional forward-biased *p*-*n* diode, where the current increases with increasing V_d due to the exponential growth of the majority carrier in the *n*-type MoS_2. Thus, the current at the positive V_d region is temperature dependent and reduces dramatically at low temperatures. At the negative V_d region, the $MoS_2/MoTe_2$ heterojunction can work as a reverse biased tunnel diode, in which the tunnelling current increases with the growth of the applied V_d. Therefore, the drain current barely changes with temperature decreases as the tunnelling current is not temperature sensitive.

Supplementary Note 5: Transfer characteristics of different fabricated transistors

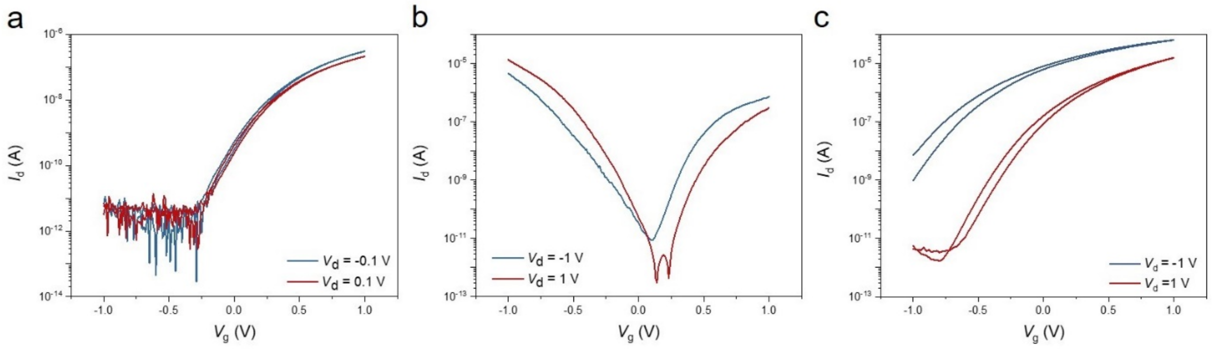

Supplementary Figure 6 | Transfer curves of MoS_2 (a) and $MoTe_2$ (b) LTFETs as well as the $MoS_2/MoTe_2$ vdW heterostructure-based VTFET (c). The device structure can be found in Figure 2b in the manuscript.

Supplementary Note 6: Discussion on the contribution of the lateral conducting channel to the overall device transport properties

It is obvious that the carrier transport path in the VTFETs consists of the lateral and the vertical directions as marked in Supplementary Figure 7. An ideal VTFET would have a minimal lateral transport channel length to offer the ultra-scaled footprint in the 2D plane, albeit that the lateral conducting line at the bottom linking the vertical channel to the source electrode is unavoidable in VTFET device structure. To address the concerns over the effect of lateral transport on the electrical properties of the VTFETs, we instead carried out simulations to tackle this issue. It is concluded that the vertical transport is the major factor determining the drain current in VTFETs as the junction resistance is higher than the lateral drift-diffusion resistance due to the presence of a *p-n* junction or a tunnelling barrier.

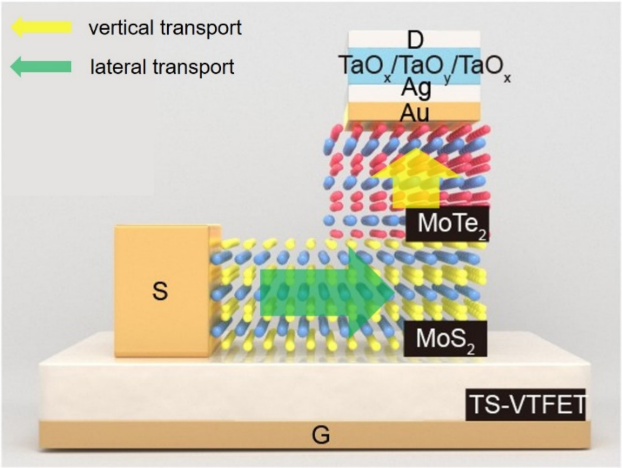

Supplementary Figure 7 | Schematic illustration of the vertical and lateral transport paths in the TS-VTFET

In the lateral direction, the length of MoS_2 is on the scale of a few microns, while in the vertical direction, the junction thickness is on the nanometer scale (~4.5 nm). The lateral transport in the VTFET proceeds by drift-diffusion with the intralayer carrier recombination within each material, while the vertical transport is dominated by the diffusion of majority carriers or the band-to-band tunnelling depending on the applied voltages. Supplementary Figure 8, 9 show the simulated band diagrams in the lateral and vertical directions of the VTFET,

respectively. At a forward bias V_d, both holes in $MoTe_2$ and electrons in MoS_2 are accumulated. Most of the voltage drop occurs across the vertical *p-n* junction, and the band bending in the lateral transport direction within each semiconductor is very small as shown in Supplementary Figure 8a. Consequently, the current in the VTFET is governed by the diffusion of majority carriers in the vertical *p-n* diode as shown in Supplementary Figure 9a, which indicates that there is a very limited effect of the lateral channel on the overall VTFET transport properties. On the other hand, under reverse bias V_d, holes in $MoTe_2$ and electrons in MoS_2 are both depleted, respectively. The depletion in the junction region and quasi-Fermi level splitting at the reverse bias would result in large band bending in the lateral direction as depicted in Supplementary Figure 8b. Meanwhile, in the vertical direction, the electrons can tunnel quantum mechanically from $MoTe_2$ to MoS_2 according to the band profile of the diode under reverse bias V_d as shown in Supplementary Figure 9b. Such a band-to-band tunnelling is well controlled by the gate voltage and is also purely dominated by the $MoTe_2/MoS_2$ vertical junction. The above analysis determines that the charge transport in VTFET is dominated by the vertical $MoS_2/MoTe_2$ *p-n* diode and has no appreciable contribution from the lateral semiconductor channel. This physics picture can be partially validated by the difference of the gate-controlled output properties between the MoS_2 LTFET and $MoTe_2/MoS_2$ VTFET (fabricated on the same flake, see Supplementary Figure 10). Clearly, under the negative gate voltage, the charge transport is dominated by the band-to-band tunnelling for the VTFET, showing a high current level under a negative drain voltage. In contrast, the MoS_2 LTFET shows no current running under this negative gate voltage, indicating a different charge transport mechanism. Therefore, benefiting from the solely vertical-junction controlled transport properties in the VTFETs, such a device has a high potential to be further scaled in the lateral direction for advanced low-footprint vertical transistors. Note that similar phenomena can be found in the atomically thin MoS_2/WSe_2 heterojunction junctions where the negligible effect of lateral transport on the atomically-thin *p-n* junction is obtained.[7]

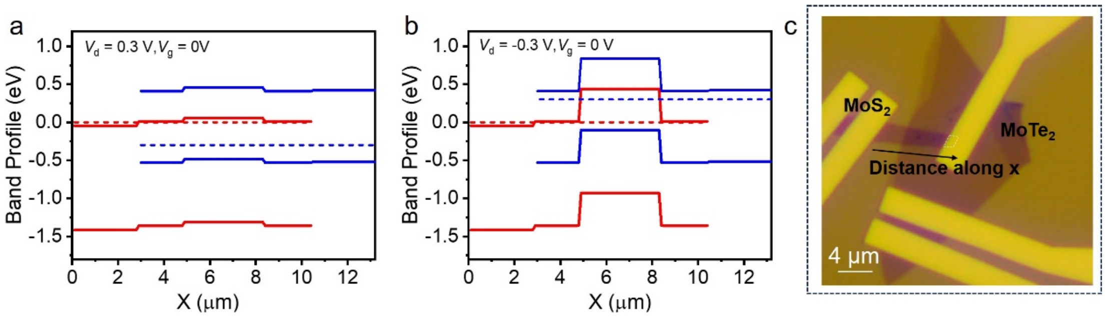

Supplementary Figure 8 | Band diagrams in the lateral transport direction of the VTFET at different V_d under V_g = 0 V. **a** V_d = 0.3 V, and **b** V_d = -0.3 V. The red (blue) dashed line represents the quasi-Fermi level of MoS_2 ($MoTe_2$). **c** Optical picture of the $MoTe_2/MoS_2$ VTFET device before capping a hBN layer.

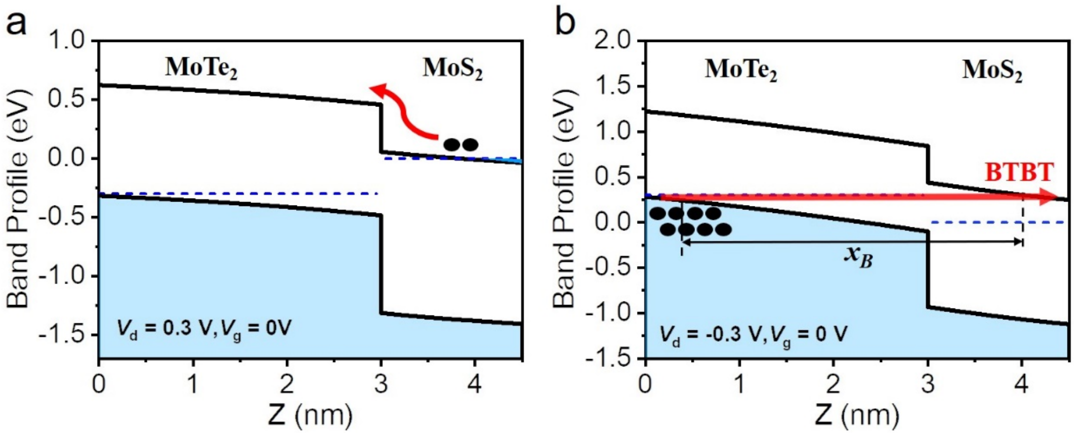

Supplementary Figure 9 | Band diagrams of the VTFET in the vertical transport direction at different V_d and V_g = 0V. **a** V_d = 0.3V, and **b** V_d = -0.3V. The dashed line represents the quasi-Fermi level.

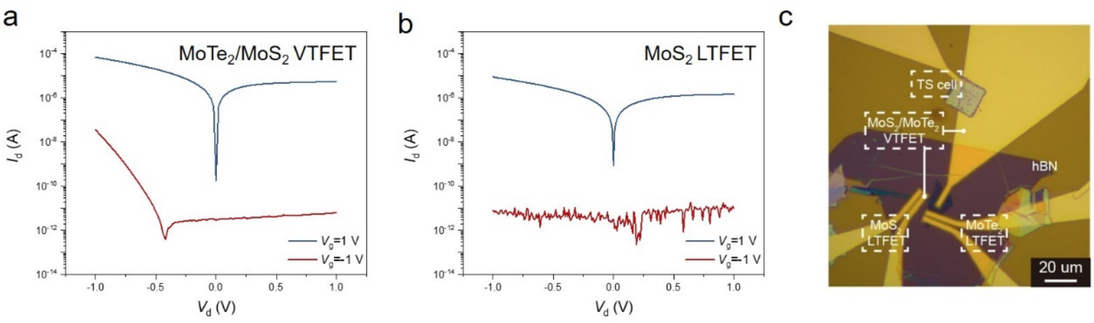

Supplementary Figure 10 | Output properties of $MoTe_2/MoS_2$ VTFET (a) and MoS_2 LTFET (b) under different gate voltages. **c** Optic photo showing the LTFET and VTFET devices fabricated with the same MoS_2 flake.

Supplementary Note 7: Discussion of the device behaviour of a VTFET with the gate electrode confined within the junction area

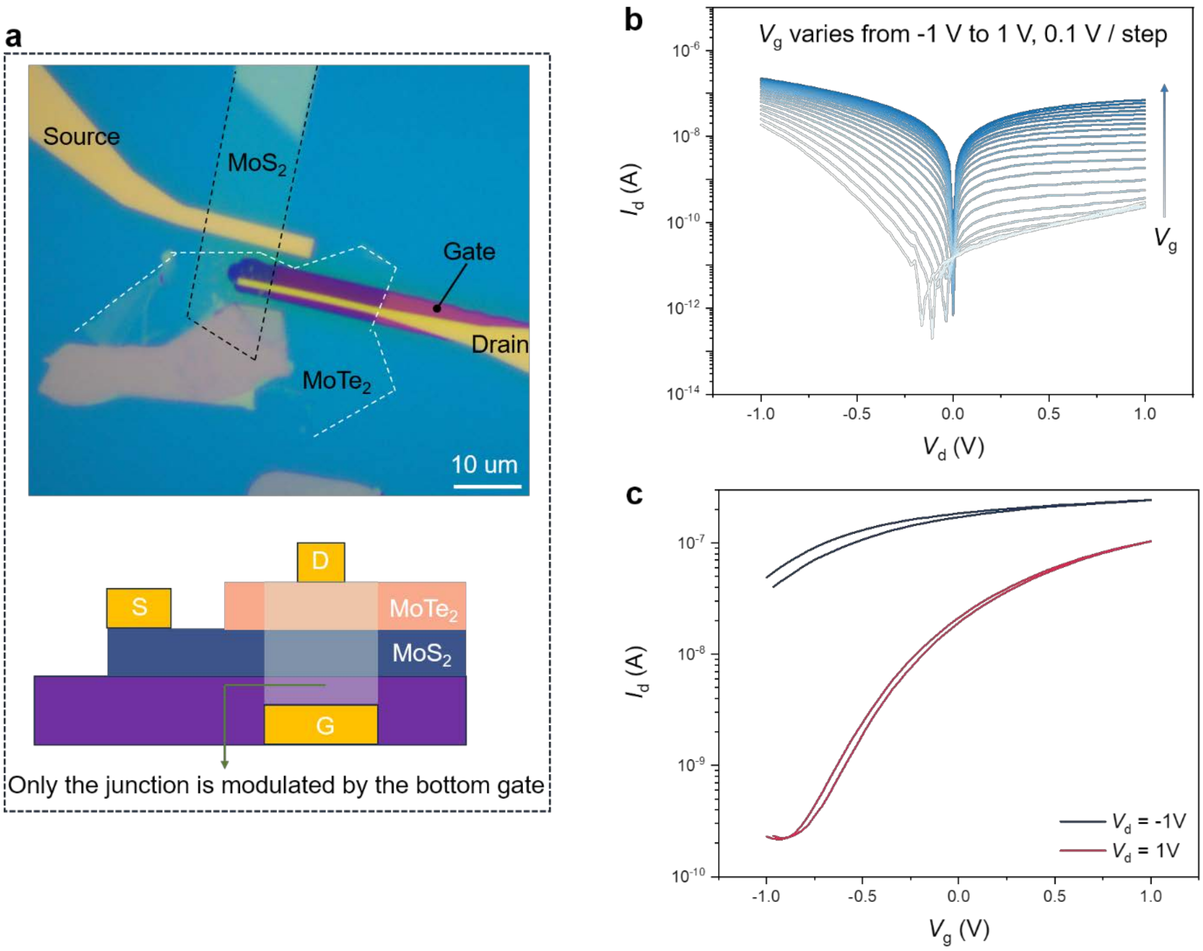

Supplementary Figure 11 | a Device structure of the $MoTe_2/MoS_2$ heterojunction VTFET with the junction area placed atop a confined back-gate electrode. The bottom gate along with the top drain electrode can only modulate the electrical properties of the overlapped $MoTe_2/MoS_2$ heterojunction. **b** Output curves at different gate voltage for $MoTe_2/MoS_2$ heterojunction VTFET. **c** Transfer curves of the VTFET measured with different V_d.

Here, we discuss that the proposed VTFET is a true vertical-transport dominated transistor. As can be seen from Supplementary Figure 11a, the drain electrode is fully confined within the bottom gate so that the gate voltage can in principle merely modulate the electrical properties of the $MoTe_2/MoS_2$ heterojunction, not affecting the MoS_2 lateral channel. With increasing the back gate voltage (V_g) from -1 V to 1 V, the rectification behaviour of output I-V_d curves

gradually disappears, see Supplementary Figure 11b. The obtained electrical behaviour is the same as the case of the $MoTe_2/MoS_2$ heterojunction VTFET with a global gate electrode, see Figure 2 of the main text. We have further measured the transfer characteristics of the fabricated VTFET (see Supplementary Figure 11c), which exhibits a clear gate-controlled OFF-to-ON conductance transition, just like the conventional MOSFET. It is obvious that the negative V_g can turn the entire device into the insulating state without affecting the MoS_2 lateral channel, thus verifying our statement that most of the V_d voltage drop occurs across the vertical *p-n* junction. With the above analysis, it would be safe to conclude that it is the $MoTe_2/MoS_2$ heterojunction that determines the transport properties of the device regardless the MoS_2 lateral channel, thus the proposed VTFET in this work is a true vertical-transport FET (VTFET). Bearing this conclusion in mind, we note that the MoS_2 lateral channel can be eliminated or largely scaled in future scalable manufacture, with the help of advanced nanofabrication techniques.

Supplementary Note 8: Microscopic structure of the Ag/TaO_x/TaO_y/TaO_x/Ag TS cell

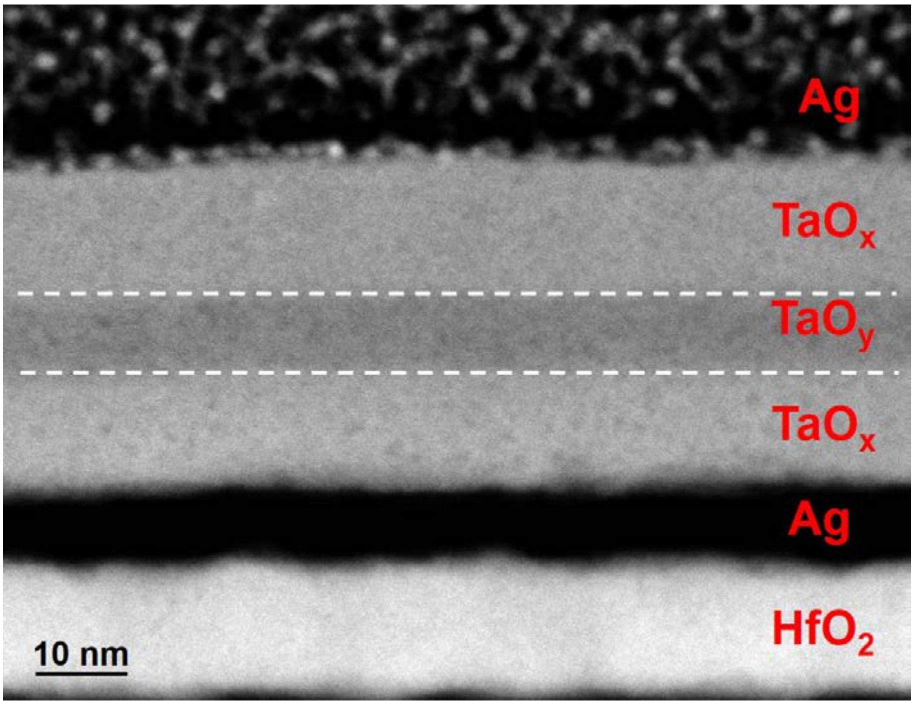

Supplementary Figure 12 | Cross-section HR-TEM image of the fabricated Ag/TaO_x/TaO_y/TaO_x/Ag TS cell. The oxygen content variation in the TaO_x and TaO_y layers can be clearly identified.

Supplementary Note 9: Electrical properties of the TS cell

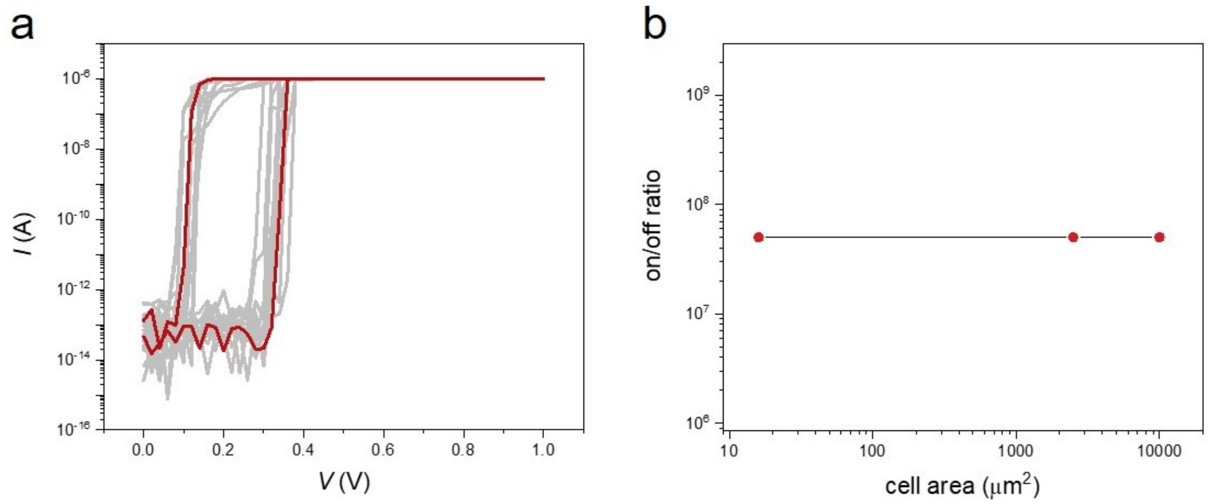

Supplementary Figure 13 | a Typical *I*-*V* curves of the $TaO_x/TaO_y/TaO_x$ TS cell with multiple consecutive switching cycles, the I_{CC} is set as 1 μA. **b** Stable switching ratio of the TS cell as a function of the cell area showing great scalability of the proposed device.

Supplementary Note 10: The electrode configuration effect on electrical properties of the TS cell

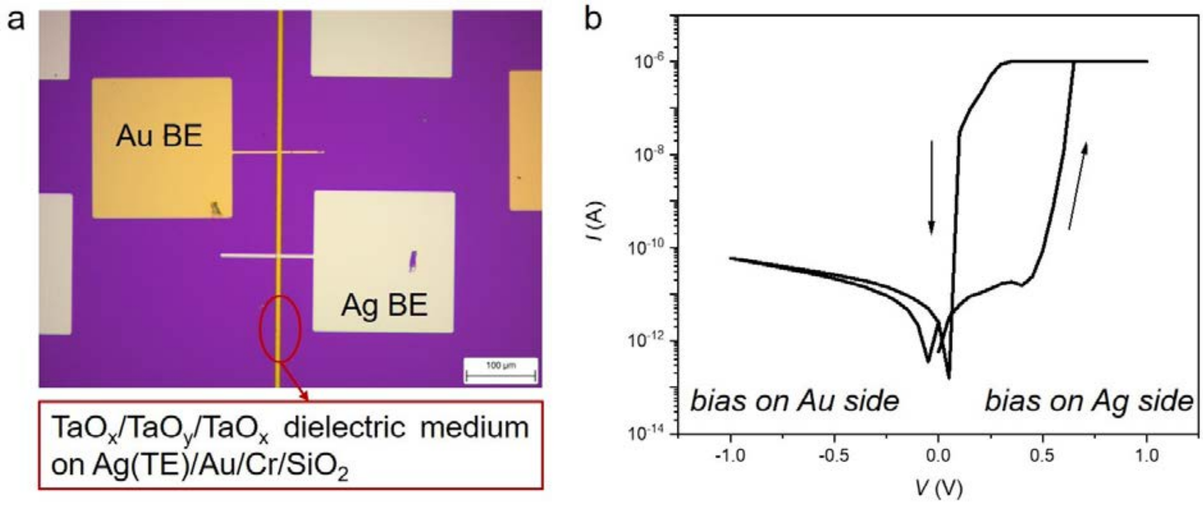

Supplementary Figure 14 | a Optical image showing the Ag/TaO_x/TaO_y/TaO_x/Au and Ag/TaO_x/TaO_y/TaO_x/Ag TS cells on the same substrate. **b** Typical unidirectional threshold resistive switching of the Ag/TaO_x/TaO_y/TaO_x/Au TS cell.

We have fabricated TS cells with both symmetric and asymmetric electrode configurations on the same substrate for comparison, i.e., Ag/TaO_x/TaO_y/TaO_x/Au and Ag/TaO_x/TaO_y/TaO_x/Ag as shown in Supplementary Figure 14. In stark contrast with the volatile bidirectional switching behaviour of Ag/TaO_x/TaO_y/TaO_x/Ag TS cells shown in the manuscript, the Ag/TaO_x/TaO_y/TaO_x/Au devices generally show unidirectional volatile switching properties. Such an Ag/TaO_x/TaO_y/TaO_x/Au cell can only go through a threshold current switching when positive voltage is applied on the chemically active Ag side and remains insulating while negative voltage is applied on the rather inert Au electrode. This is because a positive voltage applied on active Ag metal can easily trigger the growth of Ag-atom charged filaments, while a negative voltage bias on the inert Au fails to trigger the charge filament growth.[8, 9]

Supplementary Note 11: Topography of the fabricated Al_2O_3/HfO_2 substrate

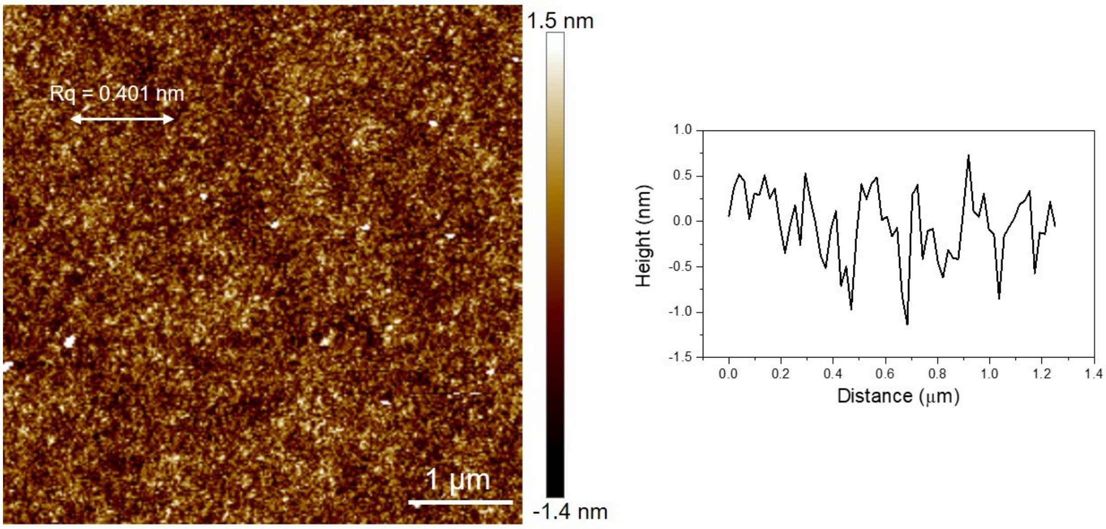

Supplementary Figure 15 | Topography and roughness of the ALD-deposited HfO_2/Al_2O_3 film.

Supplementary Note 12: Analysis of the electrical behaviours of the TS-VTFET during the current switching process

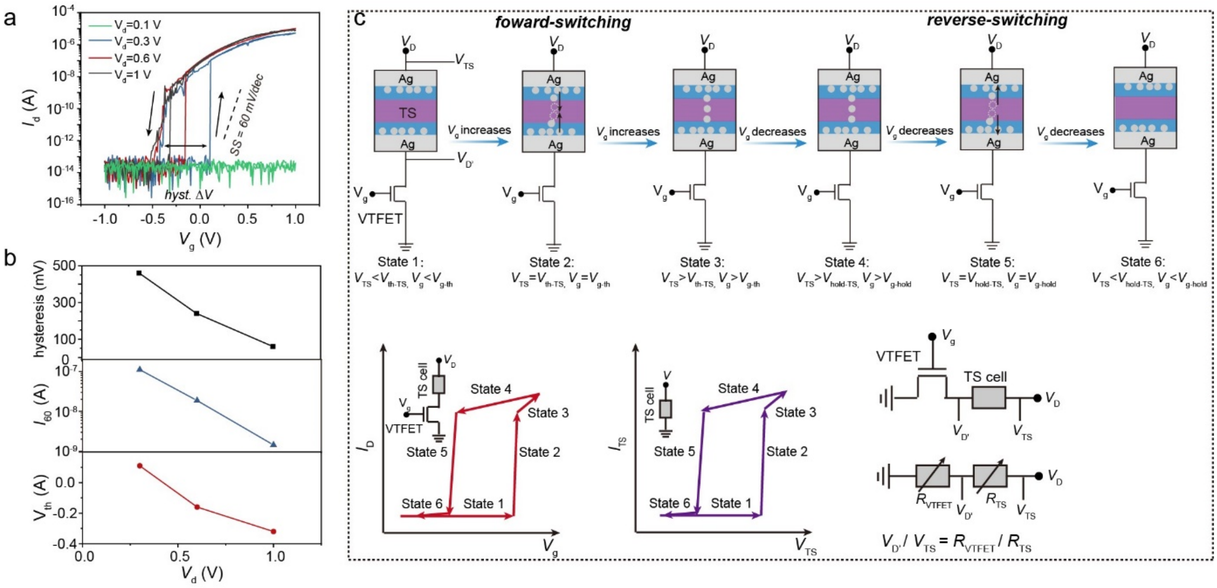

Supplementary Figure 16 | a Transfer curves of the TS-VTFET measured with different drain voltage. **b** Hysteresis voltage, I_{60} and V_{th} as a function of the applied drain voltage. **c** Schematic demonstration of the origin of hysteresis during the TS-VTFET operation.

The hysteresis in the transfer curve of TS-VTFET originates from the hysteretic current switching of the TS cell. It is evident that a hysteresis switching window exists in the IV curve of the TS cell whose width would be the voltage difference between the threshold switching and the hold point, i.e., $V_{hyst\text{-}TS} = V_{th\text{-}TS} – V_{hold\text{-}TS}$, see Supplementary Figure 16. In a TS-VTFET consisting of a TS cell and a VTFET, the two components share the supply voltage in a relation of $V_{TS} + V_{D'} = V_D$ and $V_{D'}/V_{TS} = R_{VTFET}/R_{TS}$ as shown in Supplementary Figure 16. Here, R_{VTFET} and R_{TS} can both be regarded as a variable resistor, whose resistance would be controlled by the V_g and the efficient voltage drop on the TS cell as V_{TS}, respectively. Therefore, in a hybrid system combining a TS cell and a VTFET, V_g actually controls the portion of voltage (V_D) across the TS cell through modulating the value of R_{VTFET}, which thus naturally translates the hysteresis switching of the TS cell ($V_{th\text{-}TS}$-$V_{hold\text{-}TS}$) to the hysteretic current switching in the transfer curve of the TS-VTFET ($V_{g\text{-}th}$-$V_{g\text{-}hold}$).

By choosing a V_D smaller than the V_{th} of the TS cell, even the maximum voltage

distribution with the channel part ($V_{D'}$) cannot trigger the filament formation in the case that the VTFET is fully ON. Thus, the TS-VTFET keeps at the off state as the current level is totally controlled by the TS component no matter what the V_g is, see Supplementary Figure 16a. This unambiguously proves that the TS-cell operation is controlled by both V_D and V_g. Under a fixed V_g, the VTFET should remain in a certain resistance state, the TS-VTFET could be regarded as a variable resistor (TS cell) in series with a fixed resistor (VTFET at a fixed V_g). Assuming that the R_{VTFET}/R_{TS} is almost at a constant value when applying a certain V_g on the VTFET, the efficient voltage drop on the TS component would get higher with increasing the V_D. This indicates that it is possible to trigger the threshold switching of the TS cell with a lower V_g simply by increasing the V_D, that is, the transition from state 1 to state 2-3 occurs at a lower $V_{g\text{-}th}$ (lower turn-on gate voltage for TS-VTFET). Thus, the $V_{g\text{-}th}$ gets smaller with increasing the V_D. This is because that at a high V_D, larger V_{TS} can be obtained even if the VTFET is of higher R_{VTFET} (lower V_g for *n*-type transistor), which is sufficient to trigger the threshold resistive switching of the TS component. This picture matches the observation of the relationship between V_d and V_{th} as shown in Supplementary Figure 16b. Please note that the current level of the TS-VTFET is totally controlled by the TS component at state 1. Since the hysteresis of the transfer curve (ΔV) is determined by both $V_{g\text{-}th}$ and $V_{g\text{-}hold}$, we next address the relationship between $V_{g\text{-}hold}$ and V_D. The reverse abrupt switching process of TS-VTFET is referred to state 5 with gate voltage descending (see Supplementary Figure 16c), corresponding to the hold voltage of the TS cell. Similarly, a higher V_D would contribute to a larger V_{TS}, thus the $V_{g\text{-}hold}$ of the TS-VTFET at a higher V_D is likely to be smaller compared to the case that the TS-VTFET is under a smaller V_D. Indeed, we found the experiment results of the $V_{g\text{-}hold}$ does follow the above pattern but show a small $V_{g\text{-}hold}$ difference among the cases with various V_D. In fact, the $V_{g\text{-}hold}$ shown in Supplementary Figure 16a is quite close to each other for TS-VTFET under different V_D. This may be due to the complex rupture process of the filamentary TS cells, which involves the electric field effect, Joule heating, Gibbs-Thomson effect, etc.[10]

Overall, based on the above physics picture, the improvement of hysteresis in the TS-VTFET is strongly related to the applied V_D by considering the gate voltage controlled efficient

voltage drop on the TS component. Please note that a higher V_D can lead to dramatically reduction of hysteresis but also of the I_{60} (current point where the SS becomes 60 mV/dec), which thus requires a smart trade-off between each other during practical device operation. Following the working principles stated above, to overcome the hysteresis during TS-VTFET operation, it is important to reduce the hysteresis switching window of the TS cell ($V_{th\text{-}TS}$ and $V_{hold\text{-}TS}$) itself, which thus needs a better understanding of the resistive switching in TS mediums and more advanced fabrication techniques.

Supplementary Note 13: Voltage cycling endurance of the TS-VTFETs

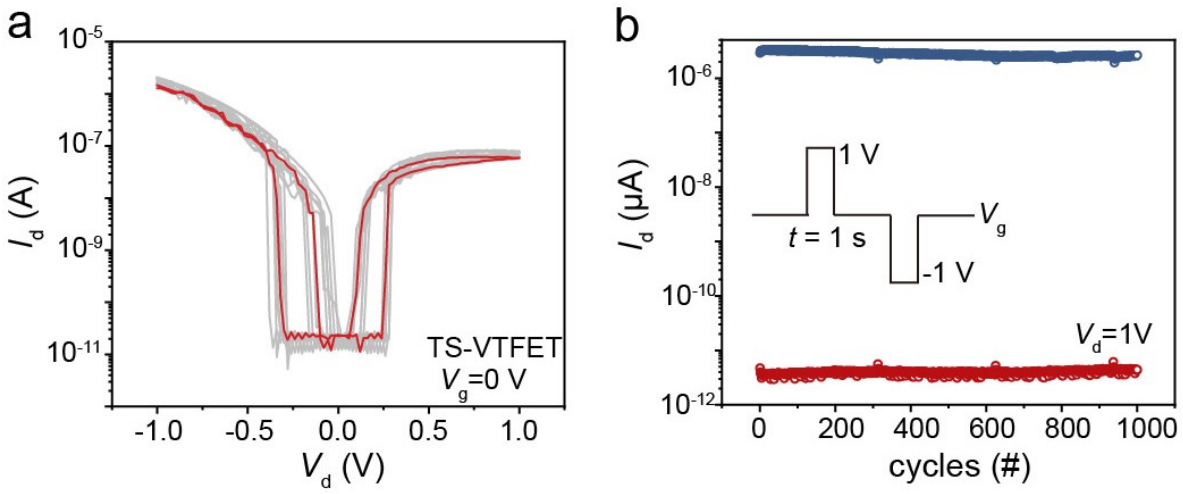

Supplementary Figure 17 | a Output curves of the TS-VTFET at Vg = 0 V for 50 cycles of drain voltage switching between -1 V and 1 V. **b** Channel current ON-OFF switching with repetitively alternating the gate voltage between -1 V and 1V, the current was recorded at V_d = 1 V.

Supplementary Note 14: Comparison of key figure-of merits of TS-VTFET with previous VTFETs

Supplementary Table 2 | Comparison of key figure-of merits of TS-VTFET with previous VTFETs.

Junction	Channel thickness (nm)	I_{ON}/I_{OFF}	I_{ON} current density (A/cm²)	V_d (V)	V_g (V)	Sub-60 SS	Ref.	comment
Gr/Si	Bulk Si	~1×10³	0.4	0.3	(-3, 3)	NO	Science 336, 1140, 2012	
Au/MoS_2/Gr	36	1.5×10³	2600	-0.5	(-60, 60)	NO	Nat. Mater. 12, 246, 2012	
	9	3.5	2100					
Ag/MoS_2/Gr (vdW metal)	3.6	~1×10³	NA	0.1	(-60, 60	NO	Nat. Electron. 4, 342, 2021	Same group, same fabrication method
	0.65	26	NA					
Ag/MoS_2/Gr (vdW metal)	5	1.85×10³	~100	0.01	(-60, 60)	NO	Nano Lett. 23, 8303, 2023	
Pt/MoS_2/Gr (vdW metal)	4.55	4.83×10⁵	~10					
Gr/WS_2/Gr	~9	~1×10⁶	~200	0.2	(-30, 30)	NO	Nat. Nanotech. 8, 246, 2012	
Au/DPA/Gr (organic SC)	~126	1.8×10⁵	~0.06	-5	(-80, 40)	NO	Adv. Mater. 30, 1803655, 2018	
Au/PDVT-10/MXene	76	2×10⁵	~0.058	-30	(-20, 10)	NO	Nat. Commun. 13, 2898, 2022	
MoS_2/$MoTe_2$	4.5	1.77×10⁸	689	0.3	(-1, 1)	YES	This work	
TFT to drive AlGaAs- and InGaN μLED			~10	NA			Nature 614, 81, 2023	ON current density needed to drive the LEDs
TFT to drive the OLED			~100	NA			Sci. Adv. 4, eaas8721, 2018	

The above table summarizes the key device parameters of representative experimental VTFETs in the literatures, to date, reporting excellent electrical properties. It is evident that out of all such reported VTFETs, the TS-VTFET is with good transistor features such as high current density, on-off ratio, sub-60 mV/dec SS behaviour, low operating voltage, etc. We also note that although our work is for realizing a new idea towards vdW heterostructure steep-slope VTFETs, the presented device already meets the current density requirement of some LED-driven TFT applications (current density > 600 A/cm^2), see Supplementary Table 2. Therefore, the proposed 2D steep-slope TS-VTFET would be practical to raise a new VTFET device concept for energy- and area-efficient transistor technology.

Supplementary Note 15: Comparison of key figure-of merits of TS-VTFET with previous reported emerging steep-slope transistors

Supplementary Table 3 | Benchmark of the device performance metrics of emerging steep-slope transistor device concepts from high-profile Nature series journals and industry-favoured conference proceedings.

material	device concept	V_d (V)	I_{ON}/I_{OFF}	I_{60}	SS_{min} (mV/dec)	SS_{avg} over 4 decades (mV/dec)	SS_{avg} over 5 decades (mV/dec)	Sub-60 region I_{60}/I_{OFF} (decades of I_d)	Ref.	comment
P-Ge/MoS_2	band-to-band Tunnel-FET	0.1	1.8×10^7	$\sim1\times10^{-9}$ A	3.8	31.1	NA	~4	Nature 526, 91, 2015	
P-Si/InSe	band-to-band Tunnel-triode	-1	$\sim1\times10^6$	$\sim1\times10^{-8}$ A/μm	6.4	34	NA	~4	Nat. Electron. 5, 744, 2022	Same group, same device structure with different 2D semiconductors
P-Si/MoS_2	heterojunction-triode	4	2×10^7	NA	> 60	NA	NA	NA	Nano Lett. 20, 2907, 2020	
$SnSe_2/WSe_2$ On HZO	NC-2D TFET	0.3	$\sim1\times10^6$	$\sim5\times10^{-10}$ A	10	55	NA	~4	Nat. Electron. 6, 658, 2023	
Si-FinFET with HZO	NC-FinFET	0.1	$\sim3\times10^6$	$\sim1\times10^{-10}$ A	~58	NA	NA	~2	Nat. Electron. 6, 390, 2023	
MoS_2 On HZO	NC-2D MOSFET	0.1	5×10^6	$\sim2\times10^{-10}$ A	17.2	NA	NA	~3	IEEE IEDM 12.4.1-12.4.4, 2020	
Ag-HfO_2 with MoS_2 FET	Filament Phase-FET	0.2	5×10^6	$\sim4\times10^{-8}$ A	2.5	3	4.5	~5	Nat. Commun. 11, 6207, 2020	
VO_2 with Si MOFET	IMT Phase-FET	1.3	$\sim1\times10^4$	$\sim7\times10^{-5}$ A	8	NA	NA	~1.3	2016 IEEE VLSI doi: 10.1109/VLSIT.2016.7573445	
Graphene/ MoS_2	Dirac source FET	0.1	$\sim1\times10^7$	$\sim1.8\times10^{-5}$ A	29	NA	NA	~3	IEEE IEDM 12.5.1-12.5.4, 2020	
$MoS_2/MoTe_2$ with TS cell	TS-VTFET	0.3	1.77×10^8	1.1×10^{-7} A	2.77	1.52	1.52	6.56	This work	

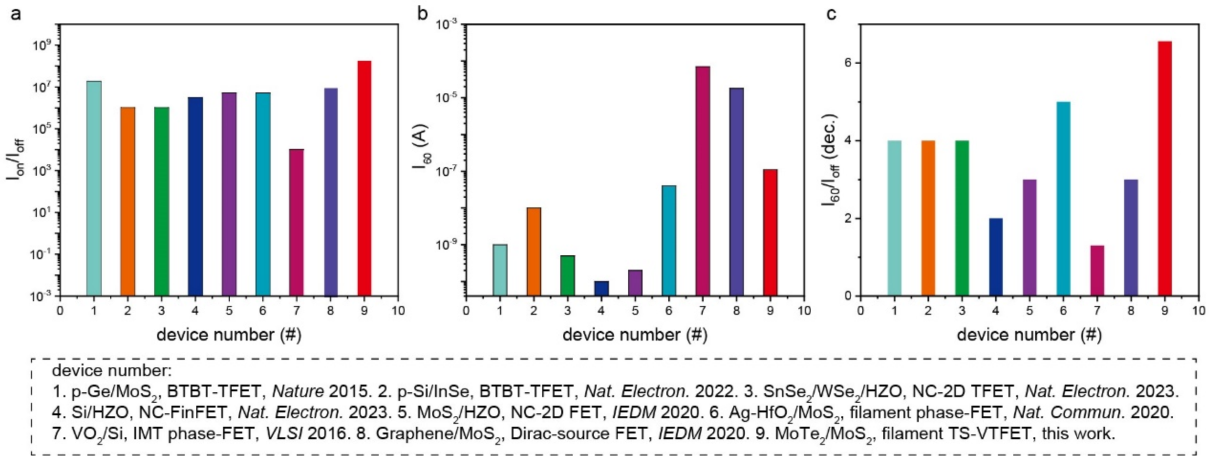

Supplementary Figure 18 | Comparison of the critical steep-slope transistor figure-of-merits for the proposed TS-VTFET with emerging steep-slope transistor device concepts. Data

extracted from Supplementary Table 3.

The proposed TS-VTFET device can achieve highly competitive individual performance metrics compared to other state-of-the-art steep-slope transistor device concepts. In Supplementary Table 3, we summarize the performance metrics of recently published high-performance steep-slope logic transistors, mainly from high-profile Nature series journals and industry-favoured conference proceedings including IEEE IEDM and VLSI. In terms of several critical performance metrics of logic transistors, the proposed device in this work shows excellent properties in comparison with other emerging device concepts including BTBT tunnel-FET (TFET), negative-capacitance (NC)-FET, NC-2D TFET, filamentary and (insulator-metal transition) IMT phase-FET, and Dirac-source injection FET. Two most important figure of merits of steep-slope devices would be the sub-60 mV/dec region and I_{60}, which determines the drain current span with SS below 60 mV/dec and the point where the current exhibits a transition from sub-60 to super-60 mV/dec.[11] Comparison of the critical steep-slope transistor figure-of-merits for the proposed TS-VTFET with other emerging steep-slope transistor device concepts is presented in Supplementary Figure 18. Normally, the IRDS mandated standard for a practical steep-slope device would require a sub-60 mV/dec region over at least 4 decades of drain current modulation and the I_{60} as high as possible. As shown in Supplementary Figure 18, the proposed TS-VTFET is among the very few devices that exhibit sub-60 mV/dec region over 4 or even 6 decades of drain current (from 10^{-14} A to 10^{-7} A). Plus, the TS-VTFET exhibits I_{60} over 1×10^{7} A, which is even comparable to the threshold drain current amplitude of most 2D MOSFETs. Thus, compared to the previous steep-slope logic transistor device concepts, the TS-VTFET presented in this work with encouraging performance metrics demonstrates a very promising solution for future high-performance steep-slope logic transistors, which is advantageous in terms of miniaturization, device performance, low power consumption and circuit design.

Supplementary References

1. Duong, N.T. et al. Modulating the functions of MoS_2/$MoTe_2$ van der Waals heterostructure via thickness variation. *ACS Nano* **13**, 4478-4485 (2019).
2. Kar, S. *High Permittivity Gate Dielectric Materials*, Springer Berlin Heidelberg (2013).
3. Wang, F. et al. Strong electrically tunable $MoTe_2$/graphene van der Waals heterostructures for high-performance electronic and optoelectronic devices. *Appl. Phys. Lett.* **109**, 193111 (2016).
4. Zhang, C. et al. Systematic study of electronic structure and band alignment of monolayer transition metal dichalcogenides in van der Waals heterostructures. *2D Materials* **4**, 015026 (2017).
5. Lam, K.T., Cao, X. & Guo, J. Device performance of heterojunction tunneling field-effect transistors based on transition metal dichalcogenide monolayer. *IEEE Electron Device Lett.* **34**, 1331-1333 (2013).
6. Chen, W., Leburton, J.P., Yin, W.Y. & Li, E. Multiphysics modeling and simulation of carrier dynamics and thermal transport in monolayer MoS_2/WSe_2 heterojunction. *IEEE Trans. on Electron Devices* **65**, 4542-4547 (2018).
7. Lee, C.H. et al. Atomically thin *p-n* junctions with van der Waals heterointerfaces. *Nat. Nanotechnol.* **9**, 676-681 (2014).
8. Lee, S., Kim, S.H., Oh, S., Lee, D. & Hwang, H. Effect of the oxygen composition control of HfO_x films on threshold and memory switching characteristics for hybrid memory applications. *Adv. Electron. Mater.* **8**, 2101257 (2022).
9. Sun, Y.M. et al. Modulating metallic conductive filaments via bilayer oxides in resistive switching memory. *Appl. Phys. Lett.* **114**, 193502 (2019).
10. Lee, J.S., Lee, S. & Noh, T.W. Resistive switching phenomena: A review of statistical physics approaches. *App. Phys. Rev.* **2**, 031303 (2015).
11. Vandenberghe, W.G. et al. Figure of merit for and identification of sub-60 mV/decade devices. *App. Phy. Lett.* **102**, 013510 (2013).
